# Role of regulatory T cells in mouse lung development

**DOI:** 10.3389/ebm.2024.10040

**Published:** 2024-03-21

**Authors:** Jian-Feng Jiang, Hong-Yan Lu, Ming-Yan Wang, Lang-Yue He, Ying Zhu, Yu Qiao

**Affiliations:** Department of Pediatrics, Affiliated Hospital of Jiangsu University, Zhenjiang, China

**Keywords:** lung development, regulatory T cells, forkhead box P3, surfactant protein C, vascular endothelial growth factor A

## Abstract

Regulatory T cells (Tregs) constitute a specialized subset of T cells with dual immunoregulatory and modulatory functions. Recent studies have reported that Tregs mediate immune responses and regulate the development and repair processes in non-lymphoid tissues, including bone and cardiac muscle. Additionally, Tregs facilitate the repair and regeneration of damaged lung tissues. However, limited studies have examined the role of Tregs in pulmonary development. This study aimed to evaluate the role of Tregs in pulmonary development by investigating the dynamic alterations in Tregs and their hallmark cellular factor Forkhead box P3 (Foxp3) at various stages of murine lung development and establishing a murine model of anti-CD25 antibody-induced Treg depletion. During the early stages of murine lung development, especially the canalicular and saccular stages, the levels of Treg abundance and expression of Foxp3 and transforming growth factor-β (TGF-β) were upregulated. This coincided with the proliferation period of alveolar epithelial cells and vascular endothelial cells, indicating an adaptation to the dynamic lung developmental processes. Furthermore, the depletion of Tregs disrupted lung tissue morphology and downregulated lung development-related factors, such as surfactant protein C (SFTPC), vascular endothelial growth factor A (VEGFA) and platelet endothelial cell adhesion molecule-1 (PECAM1/CD31). These findings suggest that Tregs promote murine lung development.

## Impact statement

Previous studies of regulatory T cells have focused on the role of Tregs in the physiological development of organs (such as the heart) and pathological repair and aberrant cell proliferation in tissues (including the lung). However, limited studies have investigated the role of Tregs during physiological murine lung development. We found that the abundance of Tregs and the expression of Foxp3 were upregulated during the canalicular and early saccular stages of murine lung development, which was aligned with the extensive proliferation of alveolar epithelial cells and vascular endothelial cells. The depletion of Tregs significantly impaired pulmonary tissue development, which was accompanied by the downregulation of lung development-related factors. This suggests that Tregs contribute to lung development.

## Highlights

• In this study, we examined the dynamic changes in Tregs and Foxp3 during various stages of mouse lung development. Subsequently, we established a mouse model with Tregs depletion to observe its impact on mouse lung development.
• We observed that Tregs and Foxp3 themselves undergo dynamic changes during lung development, and these dynamics are correlated with lung morphological changes and the pulmonary development markers SFTPC and VEGFA.
• Following Tregs depletion, we observed significant impediments in mouse lung development, including alterations in lung morphology, as well as reduced levels of SFTPC and VEGFA.
• We further explored potential mechanisms underlying the existence of these phenomena, with the hope of providing a modest theoretical basis for the study of lung development.

## Introduction

The lung develops through a series of branching morphogenesis from the endoderm. Lung development involves the gradual generation of various lineages, including airway epithelium and vascular endothelium. Lung development can be broadly categorized into the following five stages: the embryonal period, pseudoglandular stage, canalicular stage, saccular stage, and alveolar period (primarily involving the development of alveolar epithelium and pulmonary vascular endothelium) [1]. Alveolar type II epithelial cells (AEC IIs) serve as the stem cells of the alveolar epithelium. The proliferation and differentiation of AEC IIs have a critical role in alveolar development. Surfactant protein C (SFTPC), a hallmark protein expressed by AEC IIs, is an indirect marker of lung epithelium maturation [2]. Vascular endothelial growth factor A (VEGFA) is a crucial regulatory factor of pulmonary vascular development. The maturity of pulmonary vasculature can be indirectly assessed based on VEGFA expression levels [3].

Recent studies have suggested that Tregs promote cell proliferation and tissue development, Tregs with this functional profile are often characterized by the expression of CD4^+^CD25+Foxp3+ [4]. Tregs are a distinct subset of CD4^+^ T cells with immunomodulatory functions. CD25 is predominantly expressed on the surface of immune-related cells, including activated Tregs and effector T cells. CD25 blockade significantly inhibits the proliferation of Tregs, with minimal impact on effector T cells [5]. Forkhead box P3 (Foxp3), a transcriptional factor, serves as a marker of Tregs [6]. Treg depletion is reported to significantly inhibit mouse cardiomyocyte proliferation and differentiation [7]. Additionally, Treg depletion regulates the proliferation and differentiation of injured alveolar epithelial cells and impairs the generation of new blood vessels in damaged lungs [8, 9]. Conversely, Treg overexpression significantly promotes the aberrant proliferation of lung tumor cells [10]. Previous studies have focused on the role of Tregs in the physiological development of organs (such as the heart) and pathological repair and aberrant cell proliferation in tissues (including the lung), and TGF-β plays a role in Tregs regulation between tissue development [11]. However, limited studies have investigated the role of Tregs during physiological murine lung development. This study aimed to provide a theoretical foundation for lung development research by examining the dynamic changes in Tregs and Foxp3 expression at different stages of murine lung development, as well as by establishing a Treg-depleted murine model through the intraperitoneal injection of anti-CD25 antibodies, to explore the potential roles of Tregs in murine lung development.

## Materials and methods

### Experimental animals

C57BL/6J mice of similar bodyweight (22–25 g) and age (10–12 weeks) were obtained from the Animal Center of Jiangsu University (protocol No. UJS-IACUC-AP-2020030304). Three female mice were housed with one male mouse for mating. The vaginal secretion of the female mice was smeared on the next morning to examine the presence of sperm under a microscope. The day the sperms were detected was considered gestational day 0.5.

### Specimen collection and processing

Pregnant mice were anesthetized via intraperitoneal injection of 200 g/L urethane on day 17.5 of gestation. After sacrificing the mice via cervical dislocation, the abdominal cavity was rapidly exposed. The pregnant uterus was separated, and the fetal mouse was removed. The fetal lung on day 17.5 of gestation (E17.5 d, canalicular period) was collected under a microscope. On postnatal days 1 (N1 d, early saccular stage), 4 (N4 d, late saccular stage), 7 (N7 d, early alveolarization stage), 14 (N14 d, mid alveolarization stage), and 21 (N21 d, late alveolarization stage), mice at different stages of lung development were sacrificed. The lungs were harvested at the indicated time points, and the chest cavity was rapidly exposed. Both lungs were irrigated with phosphate-buffered saline (PBS, pH 7.4) until they exhibited a slightly pale color. The lung tissue was harvested and embedded for histological staining. The remaining tissues were stored at −80°C in the refrigerator for future use.

### Analysis of pulmonary tissue morphology and alveolar count

The pulmonary tissue was paraffin-embedded and sectioned into 3-µm-thick sections. The sections were subjected to hematoxylin-eosin (HE) staining. The nuclei exhibited dark blue staining, while the cytoplasm and fibrous tissue exhibited red staining. The pulmonary tissue morphology was observed under a microscope. Next, the radial alveolar count (RAC) was determined. The stained pulmonary tissue sections were placed under an optical microscope and observed at ×200 magnification. A vertical line was drawn from the center of the bronchiole to the nearest fibrotic septum or pleura. The number of alveoli along this line was counted. Five sections from each mouse were selected, and the count was repeated three times for each section to consider average value for the analysis.

### Evaluation of Pecam1 content and lung microvascular density

PECAM1 is commonly used to label lung microvascular endothelial cells [12]. The lung tissue sections were routinely dewaxed, hydrated, subjected to antigen retrieval, blocked with bovine serum albumin, and incubated with rabbit anti-Pecam1 primary antibodies (1:50), followed by incubation with goat anti-rabbit IgG secondary antibodies (1:5,000), staining with diaminobenzidine, and counterstaining with hematoxylin. Next, the section was dehydrated, cleared, and sealed. The images of Pecam1 immunohistochemical staining were analyzed using Image J. The presence of brownish-yellow regions in the cells was considered positive staining. The average optical density of the positive cells was recorded as the Pecam1 content in the lung tissue specimen. The lung microvascular density was calculated as follows: lung microvascular density (%) = (area of Pecam1-positive endothelial cells in the lung tissue/total area of lung parenchyma cells) × 100%.

### Flow cytometric analysis of the relative number of Tregs

The lung tissues were suspended in PBS and labeled with anti-FVD [detected using allophycocyanin (APC)-A750 channel] and anti-CD45 (detected using BV510 channel) antibodies to isolate living lymphocytes. Next, the cells were incubated with anti-CD4 [detected using phycoerythrin (PE) channel] and anti-CD25 antibodies (detected using APC channel) for 30 min at 4°C in the dark to stain the cell surface antigens. The cells were washed twice with PBS containing 3% calf serum. The cell membrane and nuclear membrane were disrupted using a reagent kit. Next, the cells were incubated with anti-Foxp3 nuclear antibodies [detected using fluorescein isothiocyanate (FITC) channel]. The cells were then centrifuged and washed. The CD4^+^CD25+Foxp3+ cells were considered Tregs. The flow cytometric data were analyzed using FlowJo software.

### Quantitative real-time polymerase chain reaction (qRT-PCR) analysis

The lung tissue was weighed (100 mg), transferred to a sterile mortar, and homogenized with 1 mL of Trizol reagent to extract total RNA from the tissue. The isolated RNA was reverse-transcribed into complementary DNA, which served as a template for amplification with primers, using a reverse transcription kit. The PCR conditions were as follows: denaturation at 95°C for 30 s, followed by 40 cycles of 95°C for 5 s and 60°C for 30 s. The Ct value was obtained from the Real-time RT-qPCR instrument. β-actin served as the internal reference. The ΔCt and ΔΔCt values were calculated. The relative expression levels of target mRNAs were calculated using the 2^−ΔΔCT^ method.

### Western blotting

The lung tissues were cut into small pieces and lysed in lysis buffer containing protease inhibitors. The protein samples (10 µL) were subjected to electrophoresis. The resolved proteins were transferred to a membrane. The membrane was blocked and incubated with rat anti-Foxp3 (1:1,000), rabbit anti-CD4 (1:500), rabbit anti-TGF-β (1:1,000), rabbit anti-SFTPC (1:200), and rabbit anti-VEGFA (1:1,000) antibodies overnight at 4°C. After washing, the membrane was incubated with the secondary antibodies (1:5,000) at room temperature. Immunoreactive signals were developed using chemiluminescence. The grayscale value of immunoreactive signals was quantified using Image J software.

### Establishment of the Treg-depleted mouse model

Previous studies have reported that the intraperitoneal injection of 100 µg of rat anti-mouse CD25 monoclonal antibodies at days 10 and 15 of pregnancy depletes Tregs in pregnant female mice and fetal mice [7]. Mice in the control group were injected with an equal volume of IgG isotype control antibody as a negative control for anti-CD25 antibodies. The number of CD25+Foxp3+ cells in the lung tissues was examined using flow cytometry. The expression of CD4 mRNA was detected by RT-qPCR, and the expression of CD4 protein was detected by Western blotting to evaluate the effect of Tregs depletion (Tregs belong to CD4^+^ T cells). The morphology of the lung tissue, the number of alveoli, the content of Pecam1, the density of microvessels, and the mRNA and protein expression levels of Foxp3, TGF-β, SFTPC, and VEGFA were examined in Treg-depleted mice on day 17.5 of pregnancy, day 1 after birth, and day 4 after birth and compared with those in the control group.

### Statistical analysis

Data analysis was performed using GraphPad Prism 9.0 software. Quantitative data with normal distribution are expressed as mean ± standard deviation. Means between the groups were compared using one-way analysis of variance, followed by q-test for pairwise comparisons. Pearson correlation analysis was performed to examine the correlation between two parameters. Differences were considered significant at *p* < 0.05.

## Results

### Morphological analysis of healthy developing mouse lung tissue

**FIGURE 1:**
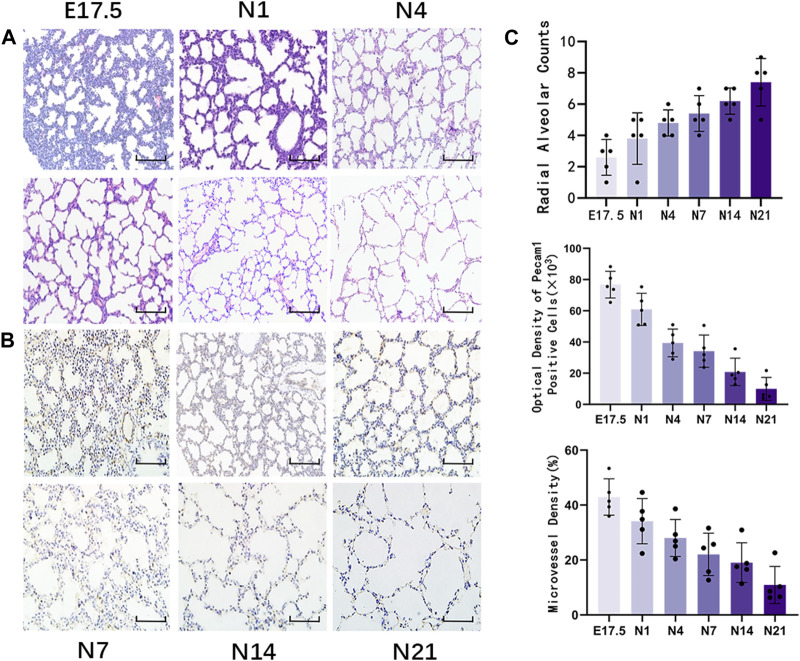
Morphological observation of healthy developing mouse lung tissue and pulmonary alveolus and microvessel counting. **(A)** The changes in alveolar structure during different stages of mouse lung development were examined using hematoxylin and eosin (HE) staining (magnification: ×200; scale bar = 50 μm, *n* = 5); **(B)** The changes in vascular structure during different stages of mouse lung development were analyzed using immunohistochemical staining (magnification: ×200; scale bar = 50 μm, *n* = 5); **(C)** Radial alveolar counts (RACs) were used to determine alveolar development. Microvascular endothelial cell development was determined based on Pecam1-labeled microvascular endothelial cells. Pulmonary microvascular maturation was determined based on the changes in the proportion of pulmonary microvascular endothelial cells to the pulmonary parenchymal area.

HE staining of lung tissues (Figure 1A) revealed visible alveolar-like structures inside the primitive lung bud at E17.5 d that were arranged in a circular pattern with tall columnar epithelial cells and dense interstitial structures. The lung buds completely separated and became independent with widely increased pulmonary alveolus spaces and comprised mostly single-layer cuboidal epithelial cells with enlarged luminal areas at N1 d. At N4 d, primary alveoli appeared with uneven sizes, thin interstitial tissues, and strip-like arrangements. The basic structural unit of the lung tissue at N7 d was primary alveoli with increased partitions and regular structures and ridges protruding into the alveoli. At N14 d, the alveoli matured with thin partitions and exhibited increased numbers. The basic structural unit of the lung tissue at N21 d was mature alveoli with regular structures and thin partitions. Quantification of the RACs at each stage of lung development (Figure 1C) revealed that the RACs gradually increased with mouse age (*p* < 0.01).

Immunohistochemical staining of Pecam1-positive cells in the lung tissue (Figure 1B) revealed that during the canalicular stage of lung development, the number of Pecam1-positive cells significantly increased and that these cells were arranged closely with the epithelium. In the same high-power microscope field, the content of Pecam1-positive cells in the pulmonary microvascular endothelial cells and the density of pulmonary microvessels were the highest. During the saccular stages, the pulmonary vessels continued to develop and the Pecam1 content and pulmonary microvascular density remained at a high level. The pulmonary vessels continued to mature, the pulmonary capillary network continued to expand, and an alveolar capillary layer that was separated from the alveolar septum formed around the mature alveoli during the alveolar period. The content of Pecam1 and the density of pulmonary microvessels continued to decrease and eventually stabilized to form a mature pulmonary microvascular network. The Pecam1 content and microvascular density in the lungs gradually decreased with the mouse age and eventually stabilized at a low level as the lungs matured (Figure 1C).

### Flow cytometric analysis of the relative number of Tregs in mouse lung tissues

**FIGURE 2:**
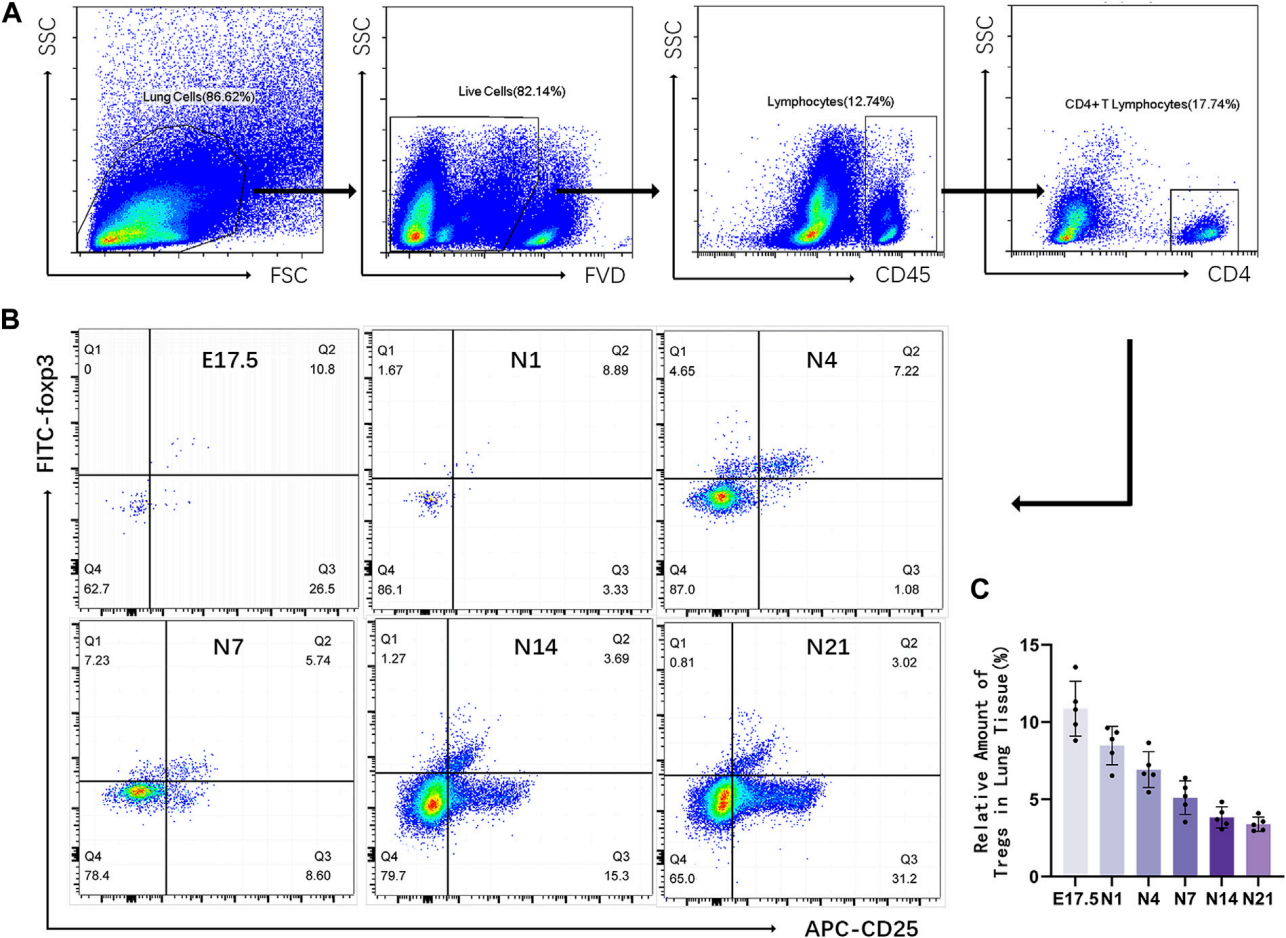
Relative number of regulatory T cells (Tregs) in the mouse lung tissue at different stages of development. **(A)** Representative results of flow cytometric analysis of Tregs in the mouse lung tissue (gated on CD4^+^CD25^+^Foxp3^+^ Tregs); **(B)** Cluster map of CD4^+^CD25^+^Foxp3^+^ Tregs at different stages of lung development; **(C)** Changes in the proportion of CD25^+^Foxp3^+^ Tregs to CD4^+^ T lymphocytes during different stages of development (*n* = 5).

Flow cytometry was used to detect CD4^+^CD25^+^Foxp3^+^ Tregs in the mouse lung tissue (Figure 2). Representative results revealed that the proportion of Tregs varied at different time points as follows: E17.5 d, approximately 10.870% ± 0.795% of CD4^+^ T cells; N1 d, approximately 8.476% ± 1.246% of CD4^+^ T cells; N4 d, approximately 6.914% ± 0.519% of CD4^+^ T cells; N7 d, approximately 5.104% ± 0.486% of CD4^+^ T cells; N14 d, approximately 3.832% ± 0.307% of CD4^+^ T cells; N21 d, 3.394% ± 0.205% of CD4^+^ T cells (Figure 1B). The pooled results revealed that the proportion of Tregs was relatively high during the canalicular stage, slightly decreased during the saccular stage, and gradually decreased with lung development during the alveolar period (*p* < 0.01) (Figure 1C).

### Analysis of Foxp3, TGF-β, SFTPC, and VEGFA mRNA and protein levels

**FIGURE 3:**
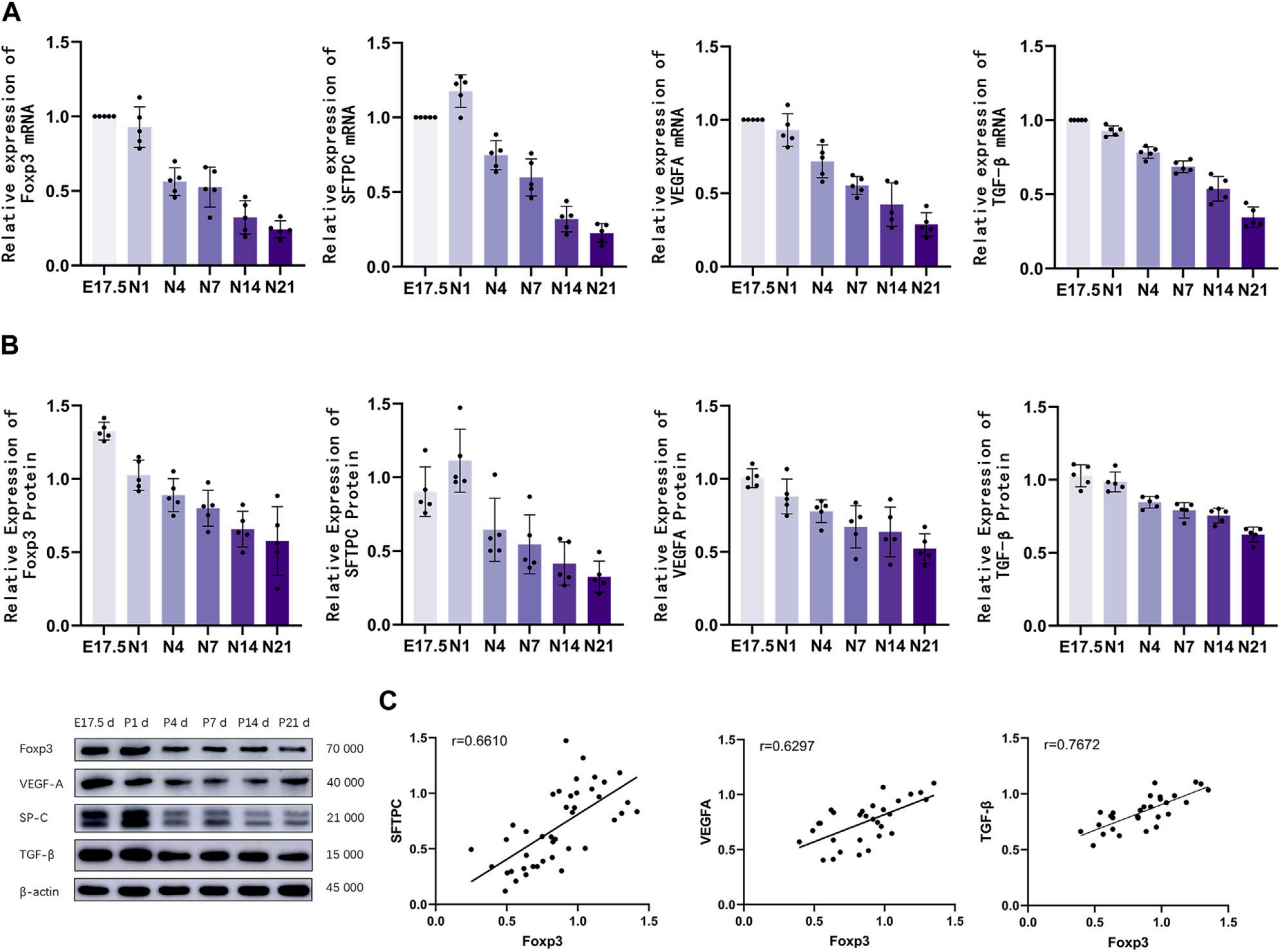
The mRNA and protein expression levels of Foxp3, TGF-β, SFTPC, and VEGFA were analyzed. **(A)** Quantitative real-time polymerase chain reaction analysis of pulmonary Foxp3, TGF-β, SFTPC, and VEGFA mRNA levels in different groups (*n* = 5); **(B)** Western blotting analysis of Foxp3, TGF-β, SFTPC, and VEGFA levels (*n* = 5); **(C)** Correlation analysis of Foxp3, TGF-β,SFTPC, and VEGFA expression levels during the development of mouse lungs.

The mRNA levels of Foxp3, TGF-β, SFTPC, and VEGFA in the lung tissues of different groups were analyzed using qRT-PCR analysis (Figure 3A). Foxp3 and TGF-β mRNA expression peaked during the canalicular stage at E17.5 d and then gradually decreased. SFTPC mRNA expression peaked on the first day after birth and then gradually decreased until the late alveolar stage. VEGFA mRNA expression peaked at E17.5 d, then gradually decreased, and finally stabilized (*p* < 0.01). The Foxp3, SFTPC, and VEGFA levels were examined using western blotting (Figure 3B). Foxp3, TGF-βwas expressed during the canalicular stages, peaked at the canalicular and saccular stages, and gradually stabilized. VEGFA expression peaked during the canalicular and saccular stages and then gradually decreased. SFTPC expression peaked on the first day after birth, then gradually decreased, and stabilized during the late stage of alveolarization (*p* < 0.01). Correlation analysis (Figure 3C) revealed that Foxp3 expression was positively correlated with SFTPC (*r* = 0.6610), VEGFA (*r* = 0.6297) and TGF-β (*r* = 0.7672).

### Establishment of the Treg-depleted mouse model

**FIGURE 4:**
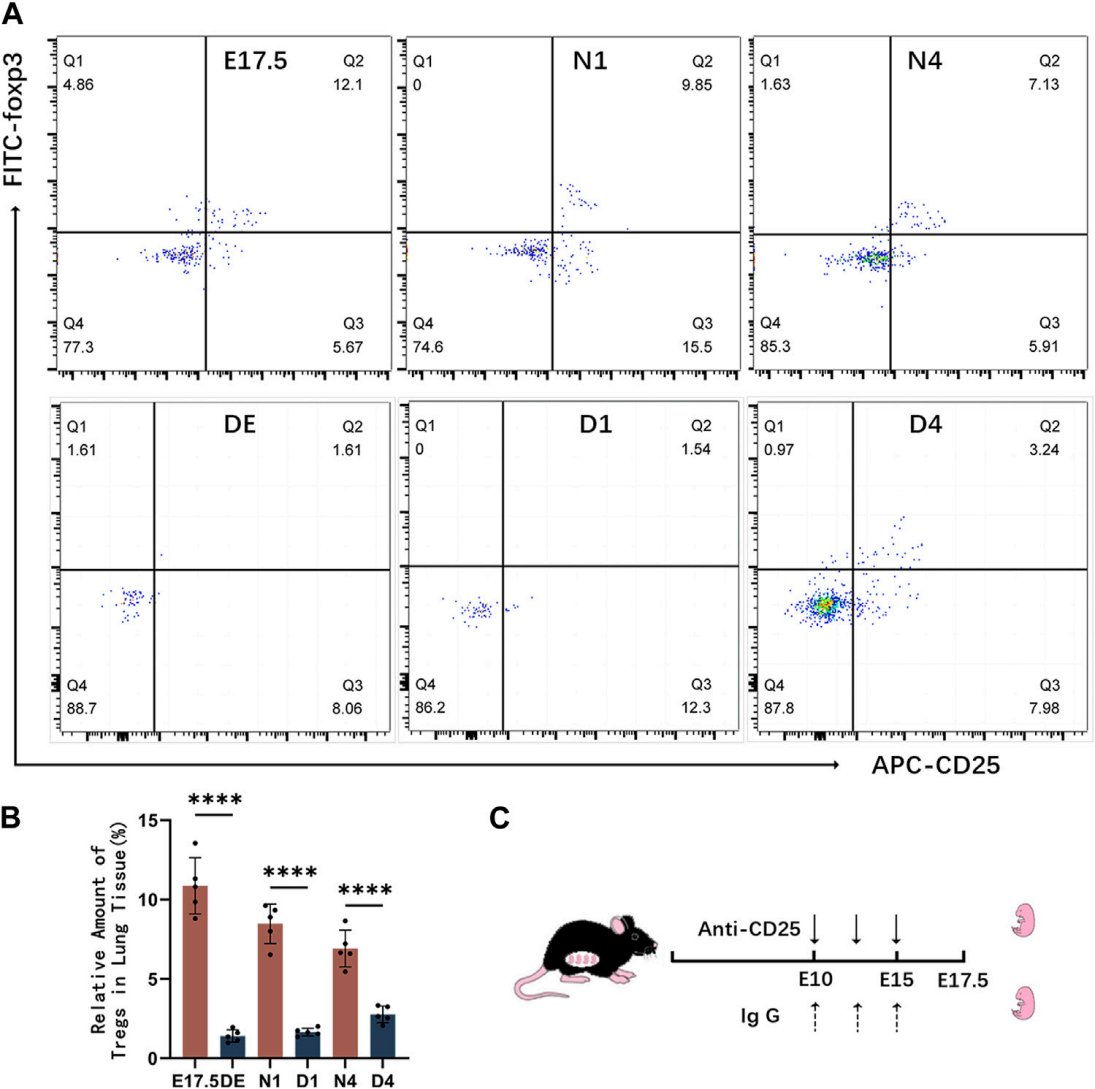
Establishment of the regulatory T cell (Treg)-depleted mouse model. **(A)** Representative results of flow cytometric analysis of CD25+Foxp3+ Tregs in the lung tissues of the control and Treg-depleted groups; **(B)** Clustering plot of CD25+Foxp3+ Tregs during prenatal lung development in the control and Treg-depleted groups; **(C)** Summary of the experimental result for Treg depletion in pregnant animals. At embryonic day 10 (E10 d) and embryonic day 15 (E15 d), pregnant mice were intraperitoneally administered with anti-CD25 neutralizing antibodies.

Mice were intraperitoneally injected with anti-CD25 antibodies to deplete Tregs. The effects of Treg depletion were examined using flow cytometry (Figure 4C). The levels of Tregs and pulmonary developmental indicators were upregulated during the canalicular and saccular stages and stabilized during the alveolar period. Hence, mice with Treg depletion were selected on gestational day 17.5 (DE group), postnatal day 1 (D1 group), and postnatal day 4 (D4 group) for comparative analysis with the control group (Figure 4A). During the canalicular and saccular stages with enhanced Treg contents, the DE and D1 groups exhibited enhanced depletion of Tregs with almost no CD4^+^ CD25+Foxp3+ T cells. Meanwhile, in the D4 group, the number of CD25+Foxp3+ Tregs slightly recovered and accounted for approximately 2.764% ± 0.235% of CD4^+^ T cells but was significantly lower than that in the N4 control group (*p* < 0.01) (Figure 4B).

### Morphological analysis of mouse lungs in the control and Treg-depleted groups

**FIGURE 5:**
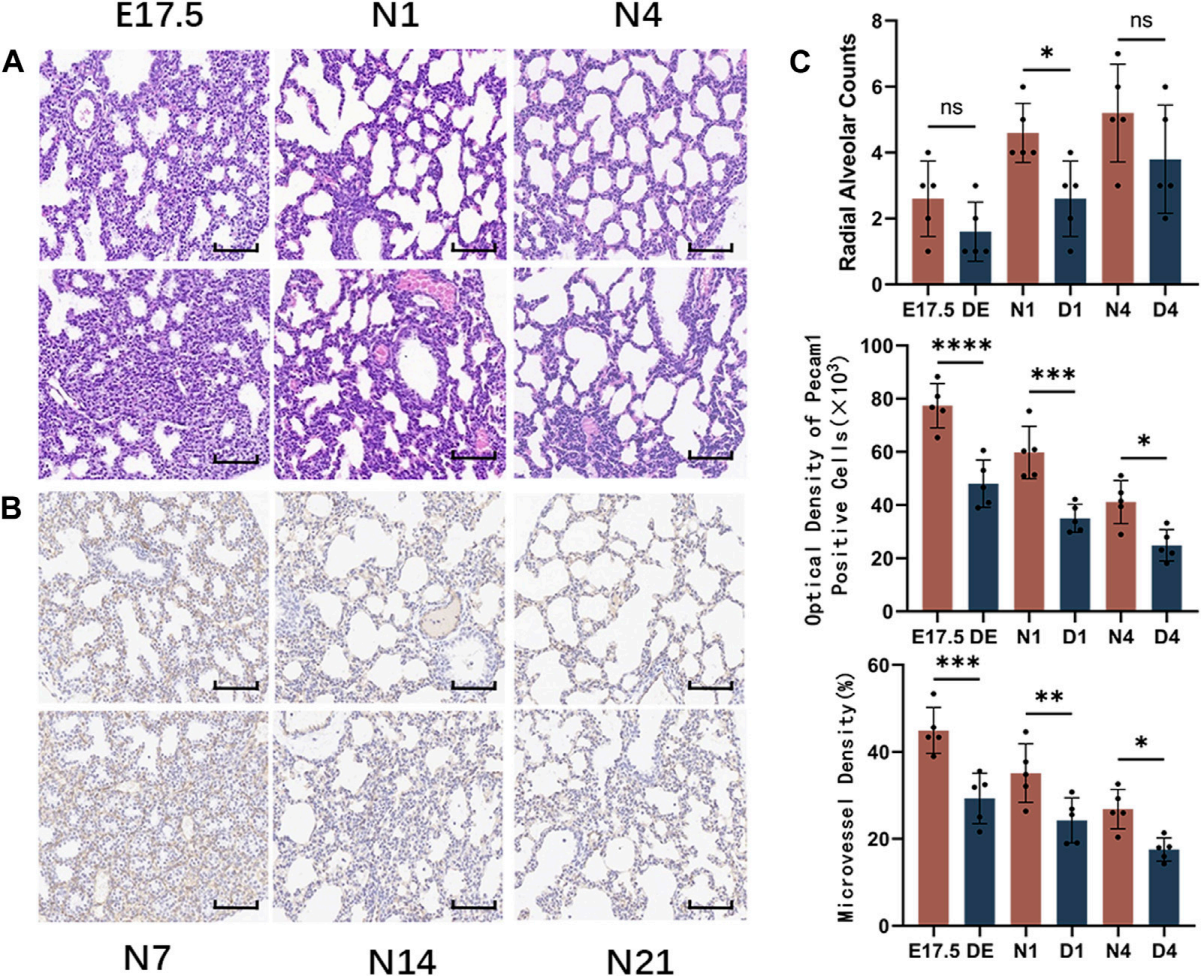
Morphological observation of mouse lungs in the control and regulatory T cell (Treg)-depleted groups during the early stage of lung development and the counting of alveolar and microvessels. **(A)** The changes in the alveolar structure during the early stage of mouse lung development in the control and Treg-depleted groups were examined using hematoxylin and eosin (HE) staining (magnification: ×200; scale bar = 50 μm, *n* = 5); **(B)** The changes in the vascular structure of the mouse lungs in the control and Treg-depleted groups during the early stage of lung development were examined using immunohistochemical staining (magnification: ×200; scale bar = 50 μm, *n* = 5); **(C)** Comparative analysis of the development of pulmonary alveoli in the control and Treg-depleted groups based on radial alveolar counts (RACs); The development of mouse microvascular endothelial cells in the control and Treg-depleted depletion groups was compared using anti-Pecam1 antibodies to label microvascular endothelial cells. Comparative analysis of the changes in the proportion of lung microvascular endothelial cells to the lung parenchyma area, which indicates the differential maturation of lung microvascular endothelial cells between the control and Treg-depleted groups.

HE staining of mouse lung tissues from the Treg-depleted and control groups (Figure 5A) revealed that compared with those in the control group, the range of airspace-like structures was significantly lower, the interstitial structure was more compact, and the number of alveolar cavities was significantly lower in Treg-depleted mice belonging to the DE group. The number of alveolar cavities was upregulated in the D1 group. However, the alveolar cavity spacing in the D1 group was thicker than that in the N1 group with uneven distribution. Only some epithelial cells were single-layer cuboidal epithelial cells with most being high columnar epithelial cells. Primary alveoli with uneven sizes were observed in the D4 group. Additionally, some alveolar cavities in the D4 group were significantly thicker than those in the N4 group. The RAC in the Treg-depleted group was significantly lower than that in the control group at N1 d. However, the downregulation of RAC at N4 d during pregnancy was not significantly different from that after birth (Figure 5C). The vascular endothelium was labeled with anti-Pecam1 antibodies (Figure 5B). The number of Pecam1-positive cells in the Treg-depleted group was significantly lower than that in the control group (*p* < 0.05). Additionally, the microvascular density in the Treg-depleted group was lower than that in the control group, especially at E17.5 d and N1 d (Figure 5C).

### CD4, Foxp3, TGF-β, SFTPC, and VEGFA mRNA and protein expression levels in the control and Treg-depleted groups

**FIGURE 6:**
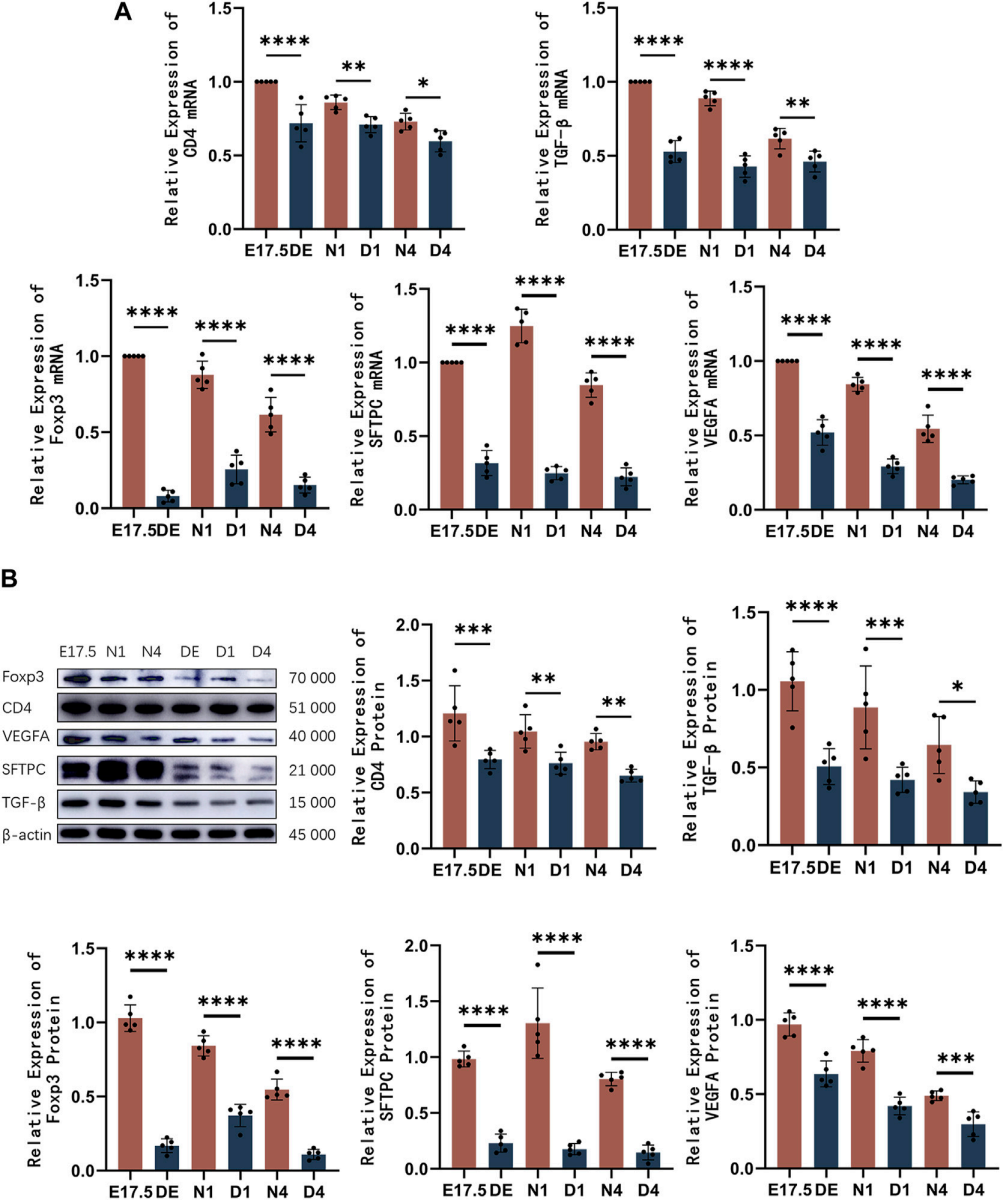
The mRNA and protein expression levels of CD4, Foxp3, TGF-β, SFTPC, and VEGFA in mice belonging to the control and regulatory T cell (Treg)-depleted groups. **(A)** Quantitative real-time polymerase chain reaction analysis of CD4, Foxp3, TGF-β, SFTPC, and VEGFA mRNA levels in mice belonging to the control and Treg-depleted groups (*n* = 5); **(B)** Western blotting analysis of CD4, Foxp3, TGF-β, SFTPC, and VEGFA protein levels in mice belonging to the control and Treg-depleted groups (*n* = 5).

The mRNA and protein levels of CD4, Foxp3, TGF-β, SFTPC, and VEGFA were analyzed using qRT-PCR and western blotting analyses, respectively (Figure 6). The expression of CD4 decreased compared to the control group. The Foxp3 and TGF-β mRNA and protein levels were significantly downregulated in the Treg-depleted group (*p* < 0.01). The SFTPC mRNA and protein levels, which peaked on day 1 after birth in mice, in the Treg-depleted group were significantly downregulated when compared with those in the control group, especially in the D1 group (*p* < 0.01). The VEGFA mRNA and protein levels were significantly downregulated in the Treg-depleted group (*p* < 0.01). The levels of these indices were not significantly different between different stages in the Treg-depleted group (*p* > 0.05).

## Discussion

In premature infants with a gestational age of less than 36 weeks, lung development is characterized by the canalicular and saccular stages until approximately 36 weeks of gestation when alveolarization begins, continuing until approximately 21 years of age. At the end of the canalicular stage, the lungs may undergo the first gas exchange, enabling the survival of extremely preterm infants [1]. The stages of mouse lung development are similar to those of human lung development. At E17.5 days, the late canalicular stage in mice is similar to that in humans (approximately equivalent to 24 weeks of gestation and alveolarization completion at approximately 36 postnatal days) [13].

The expression of SFTPC serves as an indirect indicator of alveolar epithelial cell development [14]. This study revealed that the SFTPC protein levels were upregulated at the end of the canalicular stage. After entering the mid saccular stage, SFTPC expression peaked, gradually decreased during the late saccular and alveolar stage, and finally stabilized at a low level. During the canalicular stage, the lung undergoes tubularization of the parenchyma, forming primitive alveolar ducts and sacs at the end of this stage. AEC IIs undergo proliferation at this stage, resulting in the accumulation of SFTPC protein. As the development progresses to the early saccular stage, the number of lung sacs remains relatively constant with only a few terminal airways formed during this stage. The number of AEC IIs continues to slightly increase, and SFTPC expression peaks [15]. During the late saccular and alveolar phases, most of the growth in lung volume and surface area occurs within the alveolar sacs. AEC IIs continue to differentiate and AEC Is occupy a growing portion of the area. Additionally, the SFTPC levels steadily decline and stabilize as lung development matures [16].

Vascular endothelial growth factor is one of the critical regulatory factors throughout embryonic, fetal, and postnatal lung vascular development and maintenance. VEGF exerts regulatory effects on the migration, survival, proliferation, and differentiation of pulmonary vascular endothelial cells. VEGFA is the primary structural component [17]. The expression levels of VEGFA are directly associated with the developmental state of pulmonary vasculature. Currently, limited studies have analyzed the dynamic changes in VEGFA levels during lung development. Previous studies have focused on the fetal or alveolar stages. This study revealed the dynamic alterations in VEGFA (lung vascular development-related marker) levels during lung development. During the canalicular and early saccular stages, pulmonary vascular endothelial cells undergo proliferation and differentiation and VEGFA expression is highly active. In the late saccular and alveolar stages, the rates of proliferation and differentiation of microvascular endothelial cells are delayed, which is accompanied by a gradual downregulation of VEGFA. Finally, the expression of VEGFA stabilizes. These findings are consistent with those of this study, which reported that VEGFA expression in the postnatal mouse lung gradually decreases with age [18].

Tregs, a distinct subset of T cells with both immunosuppressive and regulatory functions, are characterized by the expression of the key transcription factor Foxp3 [19]. Recent studies have suggested that Tregs exert regulatory effects on metabolism and tissue repair in non-lymphoid tissues, such as promoting the proliferation and regeneration in bone, myocardium, skin, lung, and the central nervous system [4]. This study revealed dynamic changes in the relative abundance of Tregs and the content of Foxp3 during murine lung development.

During the canalicular and early saccular stages, the contents of Tregs and Foxp3 were upregulated. At the late saccular and alveolar stages, the contents of Tregs and Foxp3 gradually decreased and stabilized during mid-to-late alveolarization. This adaptation corresponds to the extensive proliferation of alveolar epithelial cells and pulmonary vascular endothelial cells during the canalicular and early saccular stages. Correlation analysis revealed that Foxp3 expression is positively correlated with SFTPC, VEGFA and TGF-β expression, suggesting that Tregs may contribute to the development of alveolar epithelium and lung vascular endothelium. To further explore the role of Tregs during the period of extensive lung cell proliferation, a Treg-depleted mouse model was established by intraperitoneally injecting anti-CD25 antibodies. Flow cytometry revealed that the number of Tregs was significantly downregulated in the Treg-depleted group, the expression of CD4 mRNA and CD4 protein also decreased significantly after Treg-depleted, this suggests that CD25 blockade only affects Treg and has less impact on T cells. Compared with that in the control group, pulmonary alveolar development was significantly delayed in the Treg-depleted group at the same developmental stage. This delay was characterized by decreased alveolar count, alveolar septal thickening, and SFTPC downregulation. These findings suggest that Treg depletion impairs physiological pulmonary alveolar development. Limited studies have examined the role of Tregs in lung development, however, in lung injury models, Treg depletion results in the loss of regenerative capacity in damaged alveolar epithelium. Co-culturing Tregs with AEC IIs directly promotes AEC II proliferation [8]. Furthermore, Catherine F. Dial and others have demonstrated that Tregs exhibit keratinocyte growth factor (KGF) expression. Additionally, in the resolution phase of acute lung injury and the *in vivo* models of regenerating lung alveoli, Treg-specific expression of KGF promotes alveolar epithelial cell proliferation [20]. These results suggest that Tregs may directly promote pulmonary alveolar development by enhancing AEC II proliferation.

In this study, compared with those in the control group, the pulmonary levels of Pecam1 and the microvascular density were downregulated in the Treg-depleted group. Additionally, VEGFA production was downregulated, indicating that the absence of Tregs adversely affects physiological pulmonary vascular development. Tregs are involved in angiogenesis. In particular, Tregs can promote vascular formation directly by upregulating VEGFA and/or IL10 levels or indirectly by influencing other immune cells. During early lung injury repair, the depletion of Foxp3^+^ Tregs significantly suppresses pulmonary angiogenesis [21]. However, the transfer of healthy splenic cells into Treg-deficient mice promotes the full recovery of vascular generation. This suggests the role of Tregs in promoting the formation of new vessels in the lungs [22]. In this study, similar to their role in angiogenesis, Tregs may have facilitated pulmonary vascular development by directly regulating VEGFA production.

TGF-β not only influences lung development but also plays a role in T cell regulation [11]. TGF-β is required for the induction of Foxp3 in naive T cells and the development of Tregs, accordingly, Tregs also could mediate their regulation via production of TGF-β [23, 24]. Compared with those in the control group, TGF-β mRNA and protein expression levels decreased significantly in the Treg-depleted group, and there is a positive correlation between TGF-β and Foxp3 in murine lung, but the mechanisms that link Treg to TGF-β remain unknown. It is recognized that mammalian target of rapamycin (mTOR) directly links VEGF in the pulmonary branching morphogenesis program [25], threonine kinase mTOR is a critical target of TGF-β signaling in mouse [26], and mTOR signaling is a wellknown positive regulator of Tregs function under homeostasis [27]. However, further investigation is required to elucidate how CD25 blockade impacts the activation of mTOR in Tregs and lung epithelial cells. Moreover, Tregs may potentially facilitate lung development indirectly through interactions with other immune cells. Tregs can modulate development by inhibiting pro-inflammatory macrophage responses, ultimately promoting the proliferation of distal bronchioalveolar stem cells [28]. Additionally, Tregs could be attributed to the effect of macrophages and the cytokines they released in a lung ischemia mouse model. In a lung ischemia mouse model, Tregs promote angiogenesis via macrophages and the cytokines they released [22]. And Tregs may play roles in lung development by activating ILC2s, which in turn regulate homeostatic and repair processes in the lung [29].

In summary, the abundance of Tregs and the expression of Foxp3 were upregulated during the canalicular and early saccular stages of murine lung development, which was aligned with the extensive proliferation of alveolar epithelial cells and vascular endothelial cells. The depletion of Tregs significantly impaired pulmonary tissue development, which was accompanied by the downregulation of lung development-related factors. This suggests that Tregs contribute to lung development. However, the specific mechanisms and the signaling pathways involved in this process need further investigation.

## Data Availability

The original contributions presented in the study are included in the article/supplementary material, further inquiries can be directed to the corresponding author.
